# Role of platelet function testing in acute coronary syndromes: a meta-analysis

**DOI:** 10.1136/openhrt-2022-002129

**Published:** 2022-12-29

**Authors:** Anastasia Aluvilu, Albert Ferro

**Affiliations:** School of Cardiovascular and Metabolic Medicine and Sciences, British Heart Foundation Centre of Research Excellence, King’s College London, London, UK

**Keywords:** acute coronary syndrome, meta-analysis, drug monitoring

## Abstract

**Objective:**

This meta-analysis aimed to evaluate whether using platelet function testing (PFT) in acute coronary syndromes (ACS) to personalise antiplatelet therapy including a P2Y_12_ antagonist offers any clinical benefits to indicate incorporation into routine practice.

**Methods:**

A search was conducted on five databases for randomised controlled trials (RCTs) conducted between 1 January 2000 and 17 July 2022, which included an ADP-specific platelet function assays and P2Y_12_ antagonists as part of dual antiplatelet therapy (DAPT) and have reported the efficacy and/or safety outcomes. The reported event frequencies were used to calculate the risk ratios (RRs) with a 95% CI. The χ^2^ heterogeneity statistical test and sensitivity analysis were used for heterogeneity assessment.

**Results:**

Five RCTs with 7691 patients were included in the analysis. No significant risk reduction was seen in major adverse cardiovascular events (RR=0.95, p=0.42), individual cardiac events (cardiovascular death: RR=0.76, p=0.26; myocardial infarction: RR=0.96, p=0.74; stent thrombosis: RR=0.92, p=0.83; stroke: RR=0.91, p=0.72; target vessel revascularisation: RR=1.06, p=0.47) and overall clinical outcome (RR=0.90, p=0.22). There was also no difference in the rate of bleeding between PFT-guided and standard therapies (major bleeding: RR=0.97, p=0.78, minor bleeding: RR=0.89, p=0.19 and any bleeding: RR=1.04, p=0.33).

**Conclusion:**

Compared with standard DAPT with P2Y_12_ antagonists, using PFT to adjust antiplatelet therapy does not improve clinical outcomes. Therefore, the positions of key guidelines on routine testing in ACS should remain unchanged. In addition, the study highlights the need for well-designed and powered RCTs and standardised testing methodologies to provide reliable findings and definitive conclusions.

**WHAT IS ALREADY KNOWN ON THIS TOPIC**

- Platelet function testing was introduced to monitor for high on-treatment platelet reactivity during antiplatelet therapy, in order to optimise treatment. However, present guidelines do not currently recommend its routine use in the context of acute coronary syndromes treatment due to a lack of clarity on its utility.

**WHAT THIS STUDY ADDS**

- The present meta-analysis found that the application of platelet function testing during dual antiplatelet therapy including a P2Y_12_ antagonist in acute coronary syndromes does not result in better clinical outcomes.

**HOW THIS STUDY MIGHT AFFECT RESEARCH, PRACTICE OR POLICY**

- This study does not support the routine incorporation of platelet function testing into clinical guidelines for the management of acute coronary syndromes.

## Introduction

Non-communicable diseases (NCDs) are currently responsible for approximately 41 million (70%) deaths worldwide.^1 2^ Cardiovascular diseases account for 50% of these deaths, the principal cause being ischaemic heart disease as a result of atherosclerosis.^3^ Acute coronary syndromes (ACS) occurs when atherosclerotic plaques fissure or rupture within the coronary circulation, resulting in exposure of thrombogenic material and subsequent thrombosis. Platelets play a pivotal role in this process, and hence antiplatelet therapy is the cornerstone of therapy in ACS, with or without percutaneous coronary intervention (PCI).

Current treatment guidelines for ACS involve dual antiplatelet therapy (DAPT), comprising a combination of aspirin with a P2Y_12_ receptor antagonist.^4–7^ Although clopidogrel has traditionally been used as the standard P2Y_12_ receptor antagonist in DAPT, in recent years the newer agents prasugrel and ticagrelor have offered the benefits of greater antiplatelet efficacy as well as a faster onset of action,^8–11^ although they carry increased risk of bleeding complications.^12^ Additionally, approximately 30% of patients respond suboptimally to clopidogrel,^10 13–15^ largely due to slow metabolism to its active metabolite.^14 16^ Such patients, who may be identified while on clopidogrel by high on-treatment platelet reactivity (HPR), defined by persistent platelet reactivity to ADP,^17^ may theoretically derive increased benefit from one of the newer agents; but this remains to be proven.

Platelet function testing (PFT) was introduced to monitor platelet reactivity for HPR during antiplatelet therapy to optimise treatment therapy. Several PFT assays exist and have been used extensively in clinical studies. Such studies have found a clear association between HPR and the dangers of thrombotic and ischaemic complications, with a threefold early risk of ST-elevation myocardial infarction (STEMI) and a higher 6-month risk of recurrent cardiovascular events.^18–20^

Much of the existing research including meta-analyses have not categorically investigated the alteration of the DAPT regimen according to PFT in the context of ACS, despite its significant contribution to NCD deaths. However, studies that did have yielded mixed results, with some showing benefits and others none. The literature is complicated by the fact that such studies have often involved mixed populations, with the inclusion also of patients with chronic coronary syndromes and others undergoing PCI for elective reasons^16 21 22^; moreover, many of the older studies have used a mixture of different point-of-care PFT methodologies,^16 21 23 24^ although more recent studies have focused on the VerifyNow P2Y_12_ assay,^13 25–27^ which appears to give results most comparable to lab-based platelet aggregometry. Due to the lack of clarity on the utility of PFT in the context of ACS treatment, therefore, present guidelines do not currently recommend its routine use.

We aimed to assess whether PFT during DAPT including P2Y_12_ antagonists in ACS leads to better treatment outcomes, thereby justifying its integration into routine clinical practice.

## Methods

### Study design

This was a systematic review and meta-analysis (referred to as meta-analysis henceforth) looking at randomised controlled trials (RCTs) conducted between 1 January 2000 and 17 July 2022 that used PFT during DAPT with P2Y_12_ antagonists to adjust antiplatelet therapy in ACS and evaluated the efficacy or safety effects of this strategy. This meta-analysis was conducted per the Preferred Reporting Items for Systematic Reviews and Meta-Analyses 2020 statement.^28^

### Search strategy

A systematic digital search was conducted between 2 May 2021 and 17 July 2022 on the MEDLINE (via PubMed), Cochrane Library, Embase (via Ovid), Google Scholar and Web of Science engines for studies between 1 January 2000 and 17 July 2022. Suggested similar studies and those from citations of meta-analyses were retrieved. The search terms related to key concepts and their synonyms: acute coronary syndromes AND platelet function testing AND antiplatelet therapy AND P2Y_12_ antagonist AND major adverse cardiac events AND bleeding events, were tailored for each database (online supplemental table 1).

10.1136/openhrt-2022-002129.supp1 Supplementary data

### Selection criteria

Articles were screened for eligibility based on the titles, abstracts and full text in the English language to only include RCTs that compared PFT-guided antiplatelet therapy against standard DAPT in patients with ACS. The guided therapy included escalation (switching, addition, increasing dose/intensifying treatment) and de-escalation (switching from prasugrel to clopidogrel or from high-dose to low-dose clopidogrel). Furthermore, only studies that had P2Y_12_ antagonists as part of DAPT and used PFT to guide therapy were considered.

The following ADP-specific PFT assays were selected, in line with international consensus and previous studies: VerifyNow P2Y_12_ assay, multiplate analyser with ADP assay, flow cytometric assessment of vasodilator-stimulated phosphoprotein (VASP) phosphorylation index assay and conventional light transmission aggregometry assay.

Efficacy endpoints included major adverse cardiovascular events (MACE), defined as a composite of cardiovascular death, myocardial infarction (MI), both non-ST-elevation and ST-elevation types, target vessel revascularisation (TVR) and definite/probable stent thrombosis. A few studies also included endpoints of stroke or transient ischaemic attack.^29^ These cardiovascular events were also assessed individually. The safety endpoints included major bleeding, minor and any bleeding events, as per the individual study definitions using Bleeding Academic Research Consortium (BARC), Thrombolysis In Myocardial Infarction (TIMI), Global Utilization of Streptokinase and t-PA for Occluded Coronary Arteries (GUSTO) and SafeTy and Efficacy of Enoxaparin in Percutaneous coronary intervention patients, an internationaL randomized Evaluation(STEEPLE) bleeding classifications.^30–33^ Overall clinical outcome was defined as a composite of MACE and major bleeding reported in the RCTs, similar to other meta-analyses.^29 34 35^

### Study selection

Two researchers (AA and AF) independently screened the searched results by titles and abstracts. Any potentially relevant studies were retrieved with full texts for further evaluation. The two authors independently identified the eligible studies based on the eligibility criteria. The disagreements between the two authors were resolved through discussions and consensus between the two authors.

### Data extraction and quality assessment

The two researchers (AA and AF) independently extracted data from the retrieved studies using predefined variables. The following information was extracted and tabulated: first author and year of publication, total number of patients in each group (guided therapy and standard therapy), ACS clinical presentation, performance status of PCI, PFT assays and cut-off values, randomisation and PFT timepoints, type of P2Y_12_ antagonists, guided strategy (escalation and de-escalation) and follow-up duration.

### Statistical analysis

The two researchers (AA and AF) independently performed statistical analyses using the Review Manager V.5.4.1 computer package maintained by the Cochrane Collaboration.^36^ The reported event frequencies were used to calculate the risk ratios (RRs) with a 95% CI. Heterogeneity of the trials was quantified using the χ^2^ heterogeneity statistical test (p<0.05 for significant heterogeneity), with consistency assessed using I^2^, interpreted as percentages and attributed low, moderate and high in cases of 0%–25%, 50%–75% and >75%, respectively. The researchers independently performed a sensitivity analysis for outcomes with I^2^≥50%, excluding the largest study (ie, the TRANSLATE-POPS study). The researchers also independently assessed the publication bias using visual inspection of funnel plots.

## Results

### Study selection

**Figure 1:**
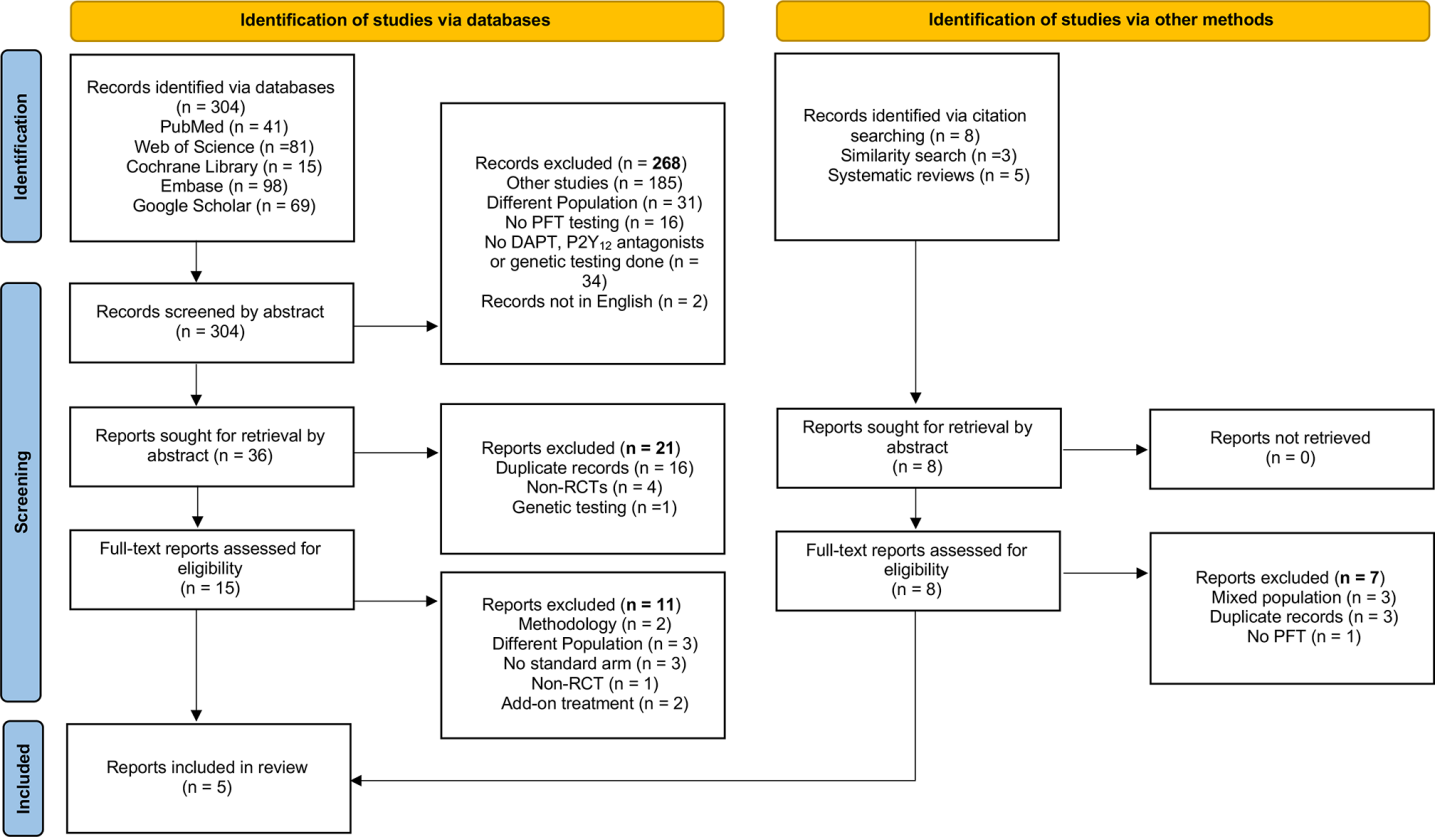
PRISMA flowchart of study selection. DAPT, dual antiplatelet therapy; PFT, platelet function testing; PRISMA, Preferred Reporting Items for Systematic Reviews and Meta-analyses; RCT, randomised controlled trial.

The search yielded 304 potentially relevant articles, and another eight were identified via citation searching (figure 1). All papers were screened at an abstract level, and 44 were then retrieved based on eligibility as judged from the abstract. Twenty-one abstracts were excluded due to duplications, non-RCTs and use of non-PFT methods. As a result, a full-text assessment was performed only on 23 papers. Although initially, seven studies met the criteria, two studies were excluded as they used add-on treatments (ie, cilostazol) as part of the PFT-guided therapy. Therefore, five studies conducted between 2014 and 2020 involving 7691 patients met the eligibility criteria and were included in the analysis.

### Study characteristics

The RCTs included showed differences in specific P2Y_12_ antagonists used, treatment strategies, sample sizes, patient characteristics, the timing of PFT, types of assays and HPR cut-off values used (online supplemental table 2). The main characteristics of the included RCTs are summarised below.

#### Treatment strategy

The type of P2Y_12_ antagonist for standard DAPT varied between the studies, either clopidogrel, prasugrel or ticagrelor as the sole P2Y_12_ antagonist or used together in some instances. Furthermore, there were differences in the loading and maintenance doses for these studies, even in instances where the P2Y_12_ antagonists used were the same. The loading doses used in the guided therapy were high-dose clopidogrel,^13 37 38^ prasugrel^25 38^ or ticagrelor.^39^ Maintenance doses included prasugrel,^13 25^ clopidogrel^13 37 38^ or ticagrelor.^39^ Two of the five RCTs used an escalation strategy,^37 39^ another used the de-escalation strategy^38^ and the other two RCTs used both strategies.^13 25^

#### Study endpoints

The study endpoints, both primary and secondary, varied considerably. For example, the primary endpoints in the PATROL study was a composite of major adverse cardio-cerebral events (MACCE) including all-cause death, cardiac death, recurrence of MI and TVR^39^; while for the TRANSLATE-POPS study,^13^ this was the frequency of ADP receptor inhibitor (ADPri) therapy adjustment during the index myocardial composite hospitalisation. Furthermore, the secondary endpoints of the TROPICAL-ACS study were BARC bleeding grade ≥2 at 12 months, stent thrombosis, incidence of any cause death and urgent revascularisation at 12 months^38^; while this included a composite ischaemic endpoint of cardiovascular death, MI, definite stent thrombosis, or urgent revascularisation at 12 months in the ANTARCTIC study.^25^

#### Sample size

Study sample sizes ranged from 87 to 3817 participants. Their calculations considered different factors such as the incidence of the primary endpoint to be 10.5% and 4.9% incidence of BARC bleeding ≥2 in the control group and an expected 45% reduction of BARC bleeding ≥2 in the de-escalation group^38^; total event rate of 19.5% for the primary endpoint and a 10% drop-out at 12 months^25^; and a 30% rate of HPR (PRU≥235) with clopidogrel in the intervention arm.^13^

#### Patient selection

The patient populations included positive biomarker ACS having undergone a successful PCI with the planned treatment of prasugrel for 12 months after the procedure^38^; STEMI-ACS patients undergoing primary PCI^39^; patients aged ≥75 years with STEMI or NSTEMI-ACS treated with PCI^25^; patients with STEMI or NSTEMI-ACS treated with PCI and ADPri therapy.^13^ While studies varied in the clinical presentation of participants, all studies focused on the ACS population.

#### Time points of randomisation

Randomisation was performed at different time points such as after PCI^13 25 37 39^ and directly before planned discharge from the hospital.^38^

#### Timing of PFT

PFT was done at least 12 hours post-PCI^13^; 12–24 hours after successful PCI^37^; 72 hours after primary PCI^39^; 14 days after randomisation and then 14 days after treatment adjustment post first stenting^25^ or 14 days postdischarge.^38^

#### Assays and HPR cut-off values

Platelet function assays and methods used to define HPR differed between studies. The multiplate analyser was used in two studies with HPR defined as ≥46 Units,^38^ while another RCT defined HPR as >46 Units and low platelet reactivity (LPR) as <19 Units.^37^ One study used VASP assay and defined HPR as PRI≥50%.^39^ Two of the studies that used the VerifyNow assay defined HPR as ≥208 PRU and LPR as ≤85 PRU^25^ and HPR as PRU ≥235.^13^

### Risk of bias assessment and publication bias

Version 2 of the Cochrane risk of bias tool for randomised trials was used and showed that all studies were overall high risk, with at least a score in one domain (online supplemental figure 1). One study had significant differences in baseline characteristics between the treatment arms,^37^ and another could potentially be affected given a higher proportion of female patients in the intervention arm.^13^ Visual inspection of the funnel plots revealed possible publication bias for cardiovascular death, stroke, MI and MACE (online supplemental figure 2).

### Effects of PFT-guided therapy on efficacy and safety endpoints

#### Major adverse cardiovascular events

**Figure 2:**
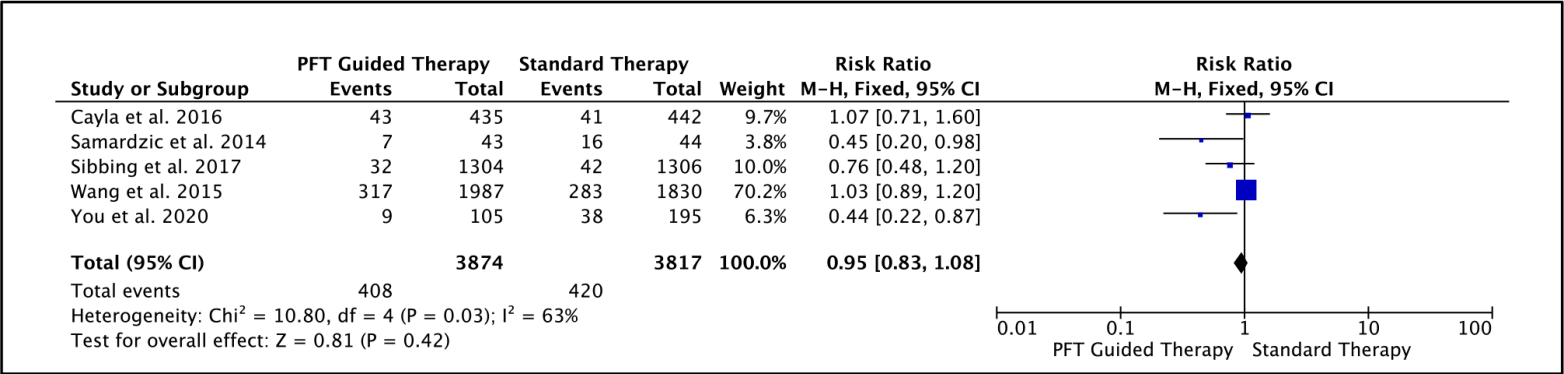
Forest plot for incidence of MACE—all assays. The risk ratios for individual studies (squares), meta-analysis (diamond) and 95% CI (horizontal lines) are presented. MACE, major adverse cardiovascular event; PFT, platelet function testing.

**Figure 3:**
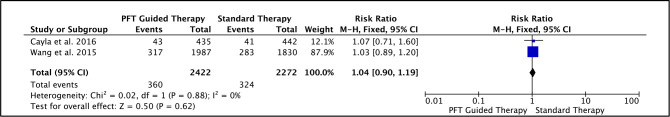
Forest plot for incidence of MACE—VerifyNow assay. The risk ratios for individual studies (squares), meta-analysis (diamond) and 95% CI (horizontal lines) are presented. MACE, major adverse cardiovascular event; PFT, platelet function testing.

PFT-guided antiplatelet therapy in general was not associated with a significant reduction in MACE (RR=0.95, p=0.42, I^2^=63% figure 2), and this was also the case with the VerifyNow assay (RR=1.04, p=0.62, figure 3). A sensitivity analysis performed excluding the largest study, TRANSLATE-POPS, also did not reveal any significant reduction in MACE (RR=0.97, p=0.64) (online supplemental figure 3). Overall, the use of PFT to guide antiplatelet therapy in ACS was not associated with a reduction in MACE compared with standard DAPT, regardless of the type of assay used.

#### Individual cardiovascular events

**Figure 4:**
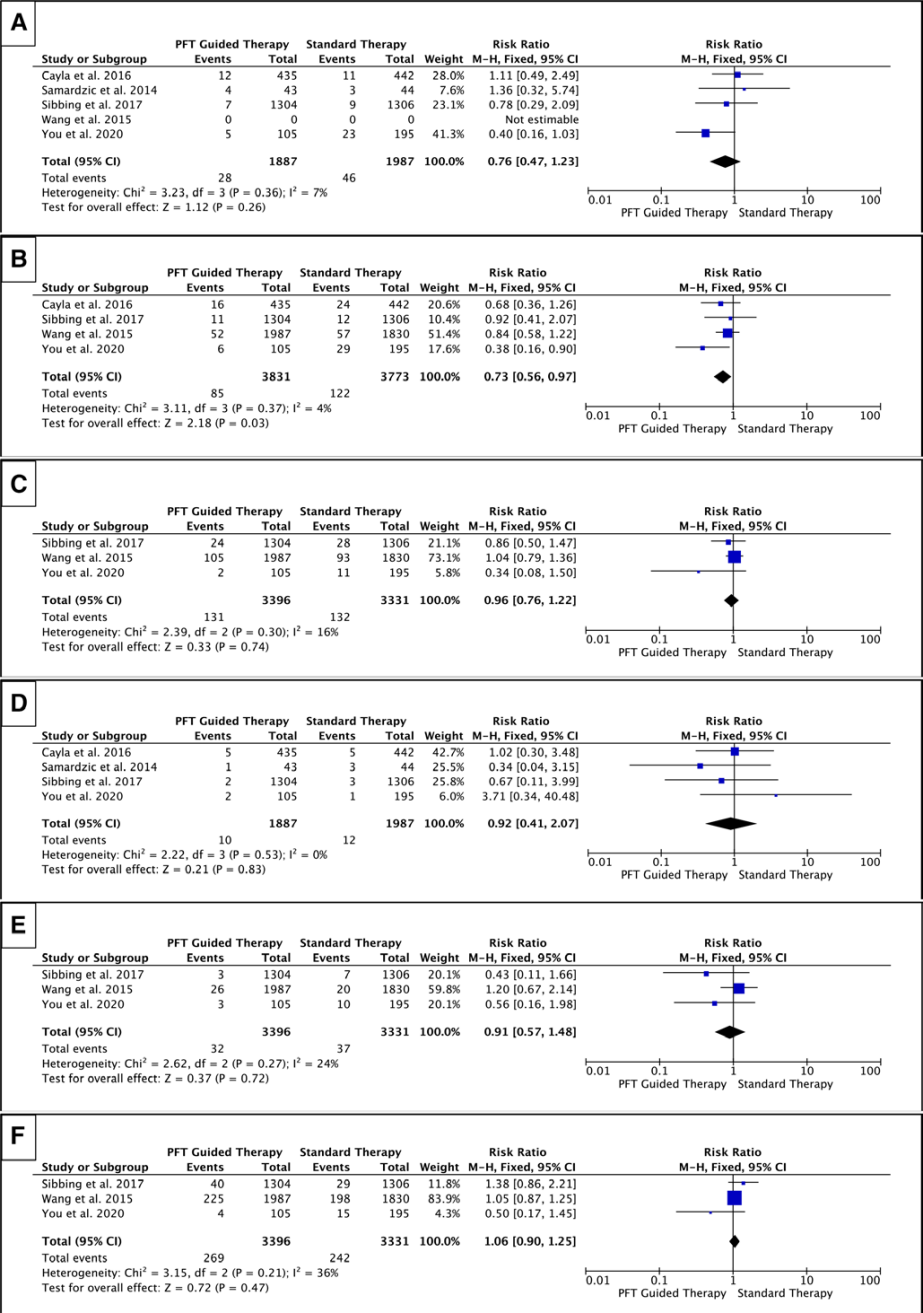
Forest plots for (A) cardiovascular mortality; (B) all-cause mortality; (C) MI; (D) stent thrombosis; (E) stroke and (F) TVR—all assays. The risk ratios for individual studies (squares), meta-analyses (diamonds) and 95% CI (horizontal lines) are presented. MI, myocardial infarction; PFT, platelet function testing; TVR, target vessel revascularisation.

**Figure 5:**
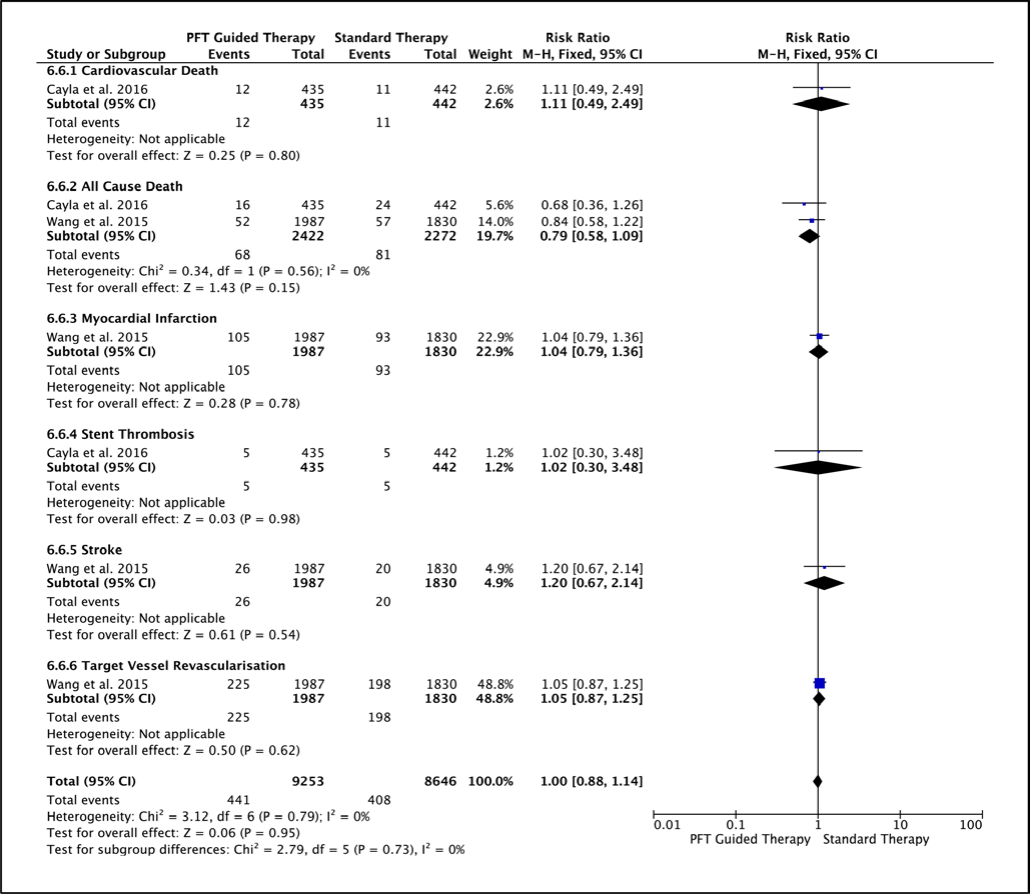
Forest plot for cardiovascular mortality, all-cause mortality, MI, stent thrombosis, stroke and TVR—specifically in studies that utilised the VerifyNow assay. The risk ratios for individual studies (squares), meta-analysis (diamonds) and 95% CI (horizontal lines) are presented. MI, myocardial infarction; PFT, platelet function testing; TVR, target vessel revascularisation.

While the PFT-guided strategy was associated with a significant reduction in all-cause death (RR=0.73, p=0.03), there was no significant reduction in other clinical events: cardiovascular death (RR=0.76, p=0.26), MI (RR=0.96, p=0.74), stent thrombosis (RR=0.92, p=0.83), stroke (RR=0.91, p=0.72) or TVR (RR=1.06, p=0.47) (figure 4). Similarly, using the VerifyNow assay did not alter cardiovascular death (RR=1.11, p=0.80), MI (RR=1.04, p=0.78), stent thrombosis (RR=1.02, p=0.98), stroke (RR=1.20, p=0.54) or TVR (RR=1.05, p=0.62) (figure 5). Therefore, these results indicate that the use of PFT to guide antiplatelet therapy in ACS does not reduce individual cardiovascular events regardless of the assay used.

#### Bleeding

**Figure 6:**
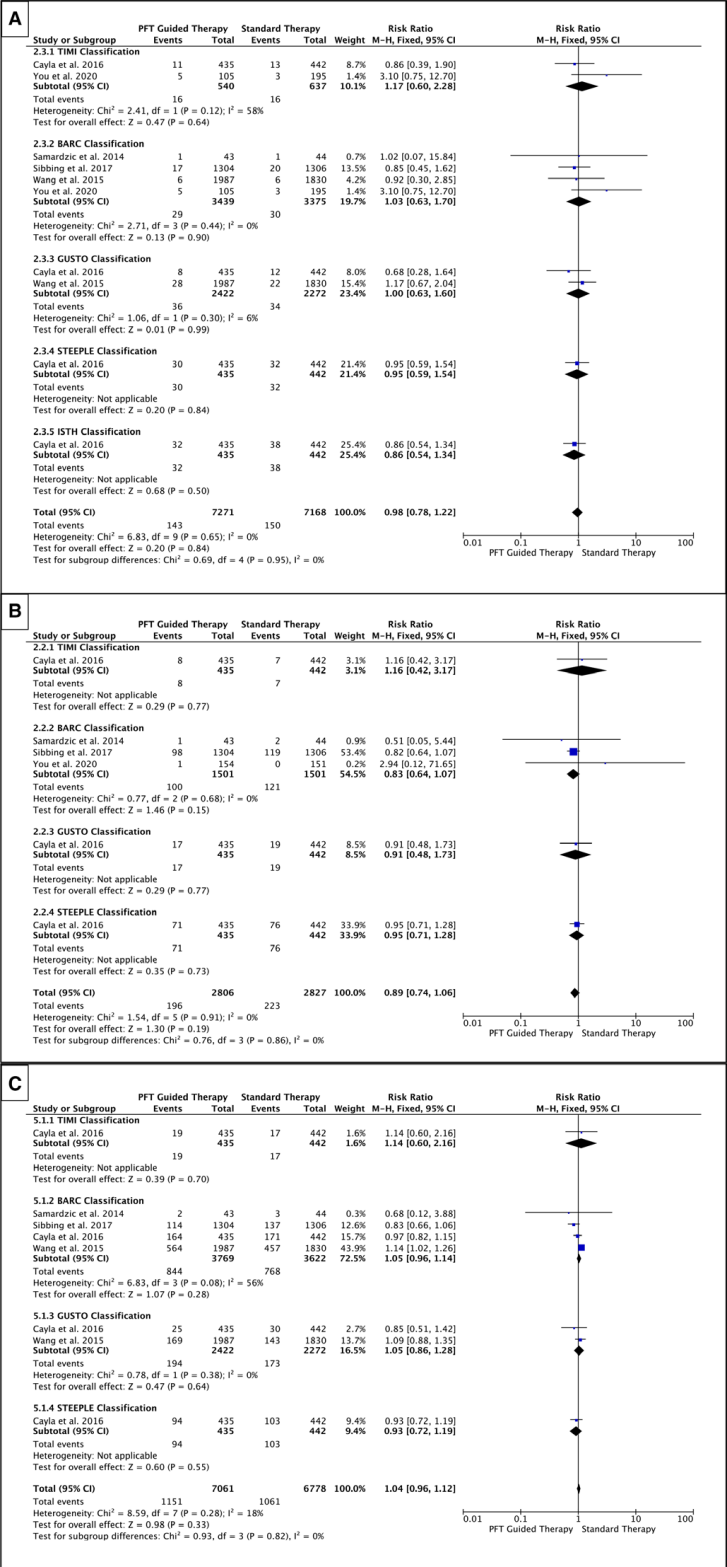
Forest plots for (A) major bleeding; (B) minor bleeding; and (C) any bleeding—all assays. The risk ratios for individual studies (squares), meta-analysis (diamonds) and 95% CI (horizontal lines) are presented. PFT, platelet function testing. TIMI, Thrombolysis In Myocardial Infarction. BARC, Bleeding Academic Research Consortium. GUSTO, Global Utilization of Streptokinase and t-PA for Occluded Coronary Arteries. STEEPLE, SafeTy and Efficacy of Enoxaparin in Percutaneous coronary intervention patients, an internationaL randomized Evaluation. ISTH, International Society on Thrombosis and Haemostasis.

**Figure 7:**
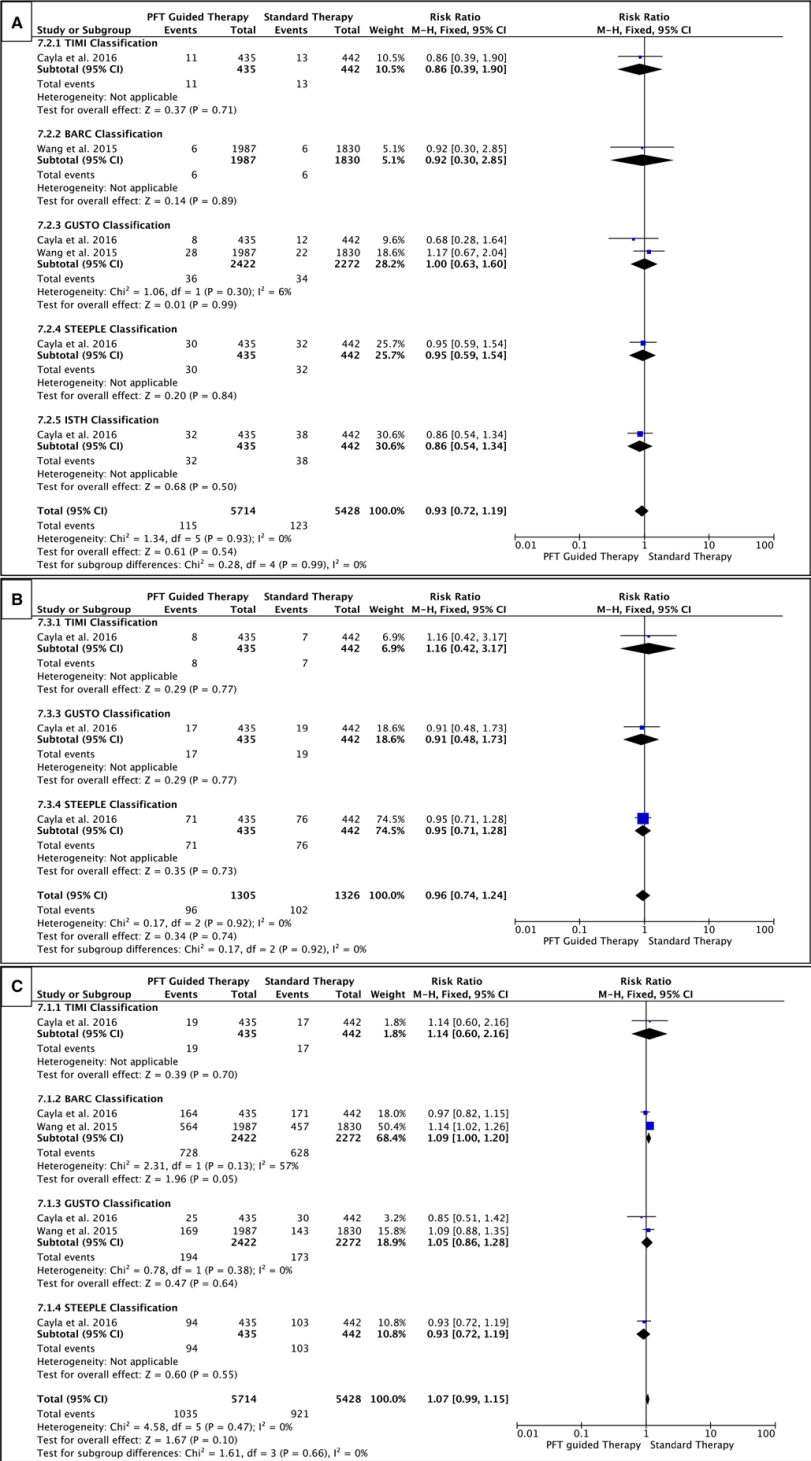
Forest plots (A) major bleeding; (B) minor bleeding and (C) any bleeding—specifically in studies that used the VerifyNow assay. The risk ratios for individual studies (squares), meta-analysis (diamonds) and 95% CI (horizontal lines) are presented. PFT, platelet function testing. TIMI, Thrombolysis In Myocardial Infarction. BARC, Bleeding Academic Research Consortium. GUSTO, Global Utilization of Streptokinase and t-PA for Occluded Coronary Arteries. STEEPLE, SafeTy and Efficacy of Enoxaparin in Percutaneous coronary intervention patients, an internationaL randomized Evaluation. ISTH, International Society on Thrombosis and Haemostasis.

Different bleeding classifications—STEEPLE, GUSTO, BARC, TIMI or two or more of these classifications—were used to assess the impact of PFT-guided therapy on bleeding (any bleeding, minor and major bleeding). Our results demonstrate that, overall, the risk of any bleeding was not different with PFT-guided therapy compared with standard antiplatelet therapy (RR=1.04, p=0.33); nor was any difference seen with major or minor bleeding (figure 6). The VerifyNow assay also showed no difference in any bleeding (RR=1.07, p=0.10) (figure 7), and similarly, no difference was seen in minor or major bleeding and the same was true of the different bleeding classifications. Therefore, using PFT to adjust therapy does not alter the risk of bleeding.

#### Overall clinical benefit

**Figure 8:**
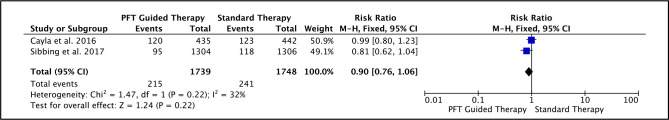
Forest plot for overall clinical outcome—all assays. The risk ratios for individual studies (squares), meta-analysis (diamond) and 95% CI (horizontal lines) are presented. The overall clinical outcome was a composite of MACE and major bleeding or MACE and bleeding of BARC ≥2. MACE, major adverse cardiovascular event; PFT, platelet function testing.

Only two studies assessed the overall clinical benefit of PFT-guided antiplatelet therapy. The results showed that there was no overall clinical benefit with PFT-guided therapy compared with standard antiplatelet therapy (RR=0.90, p=0.22; figure 8).

## Discussion

It is reported that the sample size calculation in the three pivotal RCTs, namely Gauging Responsiveness with A VerifyNow assay—Impact on Thrombosis And Safety (GRAVITAS), Testing platelet Reactivity In patients underGoing elective stent placement on clopidogrel to Guide alternative thErapy with pRasugrel (TRIGGER-PCI) and Assessment by a Double Randomization of a Conventional Antiplatelet Strategy versus a Monitoring-guided Strategy for Drug-Eluting Stent Implantation and of Treatment Interruption versus Continuation One Year after Stenting (ARCTIC) trials, were based on estimated event rates of 15%, 5% and 4.7%, respectively, and 2000–2500 patients were needed to show a significant difference.^40^ In addition, it has been suggested that studies with newer antiplatelet agents prasugrel and ticagrelor require much higher sample sizes, at least 17 000 patients, to show a significant difference.^40^ However, only two out of the five RCTs in our analysis had a sample size of at least 2000 patients. Future RCTs designed to study the effect of tailored antiplatelet therapy will need to recruit several thousand subjects to ensure that they are adequately powered.

The optimal timing of testing concerning the PCI procedure remains a topic of debate. Some studies have argued that the testing and switching should be performed early before stenting because the HPR effect is critical in the early phase.^40^ However, the time at which PFT was performed varied significantly between the different studies included in the present meta-analysis. Some studies performed PFT at least 12 hours post-PCI, and yet others performed it at 14 days and repeated it 14 days later.

A consensus has recently been reached on platelet reactivity targets, with HPR cut-off values defined as 208 PRU with the VerifyNow assay, 46 U with the Multiplate assay, 50% platelet reactivity index with the VASP assay and 47 mm with thromboelastography platelet mapping.^10 41–43^ All RCTs included in the present analysis had patients with HPR. Although these studies had used varying cut-off values and assays, efforts to optimise antiplatelet therapies with P2Y_12_ antagonists based on HPR did not reduce clinical events. The various methods used to define HPR in this analysis show that, despite having reached a consensus, there is not a defined value attached to HPR, a limitation pointed out in earlier reviews and likely to contribute to the conflicting results seen with different studies and possibly also to the negative clinical findings of this meta-analysis.^40^ This study thereby supports the view that other factors are causing negative outcomes and poor response to antiplatelet agents besides HPR.^21^ Furthermore, HPR should not be used as the sole predicting factor of clinical outcomes during antiplatelet therapy, and other strategies or combinations of strategies should be explored.

Overall, there was no significant improvement in MACE with PFT-guided antiplatelet therapy, nor did the use of PFT in guiding antiplatelet therapy have any apparent effect on bleeding. Moreover, the PFT strategy did not yield a discernible net clinical benefit. There was no detectable effect on any of these parameters when analysed either by type of P2Y_12_ inhibitor therapy or by date of study publication.

Our meta-analysis has defined MACE as a composite of cardiovascular death, MI and definite/probable stent thrombosis. However, one study used MACCE to define a composite of all-cause death, cardiac death, MI, TVR and ischaemic stroke.^39^ There were also differences in the definitions of the individual cardiac events between studies which could explain the negative results of the primary analysis.

We found that all-cause death was significantly lower in the PFT-guided group, although the absolute numbers were relatively low. The explanation for this is not clear. Effects on all-cause mortality are usually less pronounced than those on MACE because most cardiovascular studies are underpowered for the former. Interestingly, this study found the opposite, despite no apparent difference in either thrombotic or bleeding complications. It is conceivable that PFT-guided therapy reduces non-cardiac deaths, giving rise to the phenomenon. Alternatively, it is possible that a statistically non-significant reduction in MACE coupled with a small statistically non-significant decrease in bleeding give rise to an overall statistically significant reduction in mortality; however, this seems unlikely, given the lack of discernible effect on overall clinical outcomes. The effect on mortality may be a statistical anomaly, but the present finding indicates the need for further studies to examine the potential non-cardiovascular mortality benefits of PFT-guided therapy.

Differences were also seen in the bleeding definitions used. Some studies preferred the BARC, TIMI or GUSTO classification, while others reported all bleeding classifications. The use of different bleeding definitions makes therapy optimisation and interpretation for safety and efficacy comparisons difficult and could undermine the trials and the meta-analysis results in defining the safety versus efficacy balance.^33^ The BARC classification was developed in 2010 to address this problem and for adoption. However, not all studies used the BARC classification, which may explain the findings in the meta-analysis. Therefore, future RCTs should use a single standardised bleeding tool.

PFT is performed under ex vivo experimental conditions. Thus, it ignores the in vivo environment of platelets, such as the multiple stimuli acting simultaneously and the secretory role of platelets, such as the paracrine release of mediators like thromboxane A_2_ and serotonin, which might work on non-platelet targets with consequences on the clinical outcome.^44^ This makes it an inherent limitation of PFT regardless of the test used.

### Study limitations

First, no assessments were possible on the baseline characteristics on patient-level data, including age, medical and medication history (including CYP2C19-affecting medicines) to examine the relationship between these factors and treatment outcomes. This is important to determine whether drug–drug interaction, genomic and other factors affected the results due to their known contributory roles to antiplatelet therapy ineffectiveness. Second, all five RCTs included in the pooled analysis were high-risk studies with at least a score of ‘high risk’ in one bias domain. This indicates that the studies had inherent biases such as randomisation method, blinding, patient selection and reporting of results that could have affected the results. Third, this meta-analysis may have underestimated the overall clinical benefit of PFT, since only two out of five studies assessed and reported this outcome. Fourth, a meta-regression was not performed to study the impact of theoretical factors on the results, including clinical presentation (STEMI, NSTEMI or UA), platelet function assays, and P2Y_12_ antagonists. At least 10 studies are required to test one covariate in a meta-regression for adequate statistical power.^30^ While the importance of a meta-regression cannot be overemphasised, the results would still not have been powered as only five studies were included in the meta-analysis. Finally, the heterogeneity in the RCT study designs, including sample sizes, the definition of endpoints, strategies used to overcome HPR, assays and cut-off targets, may well have affected the results. Because of the differences in these components, the meta-analysis could not use standardised terms for the outcomes but relied on definitions for individual studies, for example, MACE and bleeding events (BARC, GUSTO, TIMI). Although recently published trials have done much to standardise the methodology as compared with older trials, we are not aware of any large RCTs that are currently ongoing to definitively address the limitations around adequate powering.

## Conclusion

To our knowledge, ours is the first meta-analysis to explicitly investigate the role of PFT in ACS management with DAPT including P2Y12 antagonists. We have found that the existing evidence does not support that PFT during DAPT to adjust therapy in ACS improves risks of MACE, individual cardiac events or bleeding events (major, minor or any bleeding). Additionally, the VerifyNow assay had similar clinical outcomes compared with a pooled assessment of all assays; and exclusion of VerifyNow from all assays did not change this finding. Therefore, routine PFT in ACS and its inclusion in treatment guidelines cannot be recommended based on currently available evidence. Future RCTs, as well as being adequately powered, should use standardised study designs, including the type of PFT and HPR cut-off values, endpoints, times of randomisation and testing, and definitions of safety and efficacy outcomes.

## Data Availability

All data relevant to the study are included in the article or uploaded as online supplemental information.
