# Supplementary material for: Role of platelet function testing in acute coronary syndromes: a meta-analysis
Source: Open Heart. 2022 Dec 29;9(2):e002129. doi: 10.1136/openhrt-2022-002129 (PMC9806016; doi:10.1136/openhrt-2022-002129)
Supplement: Supplementary data [file openhrt-2022-002129supp001.pdf]

SUPPLEMENTAL MATERIAL

The role of platelet function testing in acute coronary syndromes: a meta-analysis

Anastasia Aluvilu

Albert Ferro

School of Cardiovascular Medicine and Sciences, British Heart Foundation Centre of Research Excellence, King's College London, London, UK

Short title: Platelet function testing in acute coronary syndromes

Correspondence: Professor Albert Ferro. King's College London, Franklin-Wilkins Building, 150 Stamford Street, London SE1 9NH, UK. **Email:** albert.ferro@kcl.ac.uk

Supplementary Table 1. Study search terms used for digital search on different search engines.

Search engine	Search terms used
PubMed	((((("Acute Coronary Syndrome"[Mesh] OR "Myocardial Infarction"[Mesh] OR "Acute Coronary Syndrome"[tw] OR ACS[tw] OR "Non-ST Myocardial infarction"[tw] OR "ST Myocardial infarction"[tw] OR "unstable angina"[tw] OR "Non-ST MI"[tw] OR "ST MI"[tw]) AND ("Platelet Function Tests"[Mesh] OR "Platelet Function Testing"[tw] OR "Platelet Function Test*"[tw] OR PFT[tw] OR "VerifyNow"[tw] OR "Platelet Function Analyzer-100"[tw] OR "Light Transmittance Aggregometry"[tw] OR "Vasodilator-Stimulated Phosphoprotein Phosphorylation Assay"[tw] OR "Vasodilator-Stimulated Phosphoprotein Phosphorylation Assay"[tw] OR "Thromboelastography/Thromboelastometry"[tw])) AND ("Anti-platelet therapy"[tw] OR "antiplatelet therapy"[tw] OR "anti-platelet treatment"[tw] OR "antiplatelet treatment"[tw] OR "dual anti-platelet therapy"[tw] OR "dual anti-platelet treatment"[tw] OR "dual antiplatelet drug*"[tw] OR "standard antiplatelet therapy"[tw] OR "anti-platelet drug*"[tw] OR "antiplatelet drug*"[tw])) AND ("Purinergic P2Y Receptor Antagonist*"[Mesh] OR "P2Y12 receptor antagonist*"[tw] OR "P2Y12 antagonist*"[tw] OR "P2Y12 inhibitor*"[tw] OR Clopidogrel[tw] OR Ticagrelor[tw] OR Prasugrel[tw])) AND ("Cardiovascular System"[Mesh], ("Cardiovascular System/adverse effects"[Mesh] OR "Cardiovascular System/complications"[Mesh] OR "Cardiovascular System/drug effects"[Mesh] OR "Cardiovascular System/pathology"[Mesh] OR "Cardiovascular System/pharmacology"[Mesh] OR "Cardiovascular System/physiology"[Mesh] OR "Cardiovascular System/physiopathology"[Mesh] OR "Cardiovascular System/therapy"[Mesh]) OR "Major adverse cardiovascular event*"[tw] OR MACE[tw] OR "cardiovascular death"[tw] OR stroke[tw])) AND (("Hemorrhage/blood"[Mesh] OR "Hemorrhage/drug effects"[Mesh] OR "Hemorrhage/drug therapy"[Mesh] OR "Hemorrhage/pharmacology"[Mesh] OR "Hemorrhage/therapy"[Mesh]) OR "Bleeding event*"[tw] OR bleeding[tw] OR hemorrhage[tw] OR haemorrhage[tw] OR "high on-treatment platelet reactivity"[tw])
Web of Science	(("Purinergic P2Y Receptor Antagonist*" OR "P2Y12 receptor antagonist*" OR "P2Y12 antagonist*" OR "P2Y12 inhibitor*" OR Clopidogrel OR Ticagrelor OR Prasugrel) AND ("Hemorrhage/blood" OR "Hemorrhage/drug effects" OR "Hemorrhage/drug therapy" OR "Hemorrhage/pharmacology" OR "Hemorrhage/therapy" OR "Bleeding event*" OR bleeding OR hemorrhage OR haemorrhage OR "high on-treatment platelet reactivity") AND ("Cardiovascular System" OR "Cardiovascular System/adverse effects" OR "Cardiovascular System/complications" OR "Cardiovascular System/drug effects" OR "Cardiovascular System/pathology" OR "Cardiovascular System/pharmacology" OR "Cardiovascular System/physiology" OR "Cardiovascular System/physiopathology" OR "Cardiovascular System/therapy" OR "Major adverse cardiovascular event*" OR MACE OR "cardiovascular death" OR stroke) AND ("Anti-platelet therapy" OR "antiplatelet therapy" OR "anti-platelet treatment" OR "antiplatelet treatment" OR "dual anti-platelet therapy" OR "dual anti-platelet treatment" OR "dual antiplatelet drug*" OR "standard antiplatelet therapy" OR "anti-platelet drug*" OR "antiplatelet drug*") AND ("Platelet Function Tests" OR "Platelet Function Testing" OR "Platelet Function Test*" OR PFT OR "VerifyNow" OR "Platelet Function Analyzer-100" OR "Light Transmittance Aggregometry" OR "Vasodilator-Stimulated Phosphoprotein Phosphorylation Assay" OR "Vasodilator-Stimulated Phosphoprotein Phosphorylation Assay" OR "Thromboelastography/Thromboelastometry") AND ("Acute Coronary Syndrome" OR "Myocardial Infarction" OR "Acute Coronary Syndrome" OR ACS OR "Non-ST Myocardial infarction" OR "ST Myocardial infarction" OR "unstable angina" OR "Non-ST MI" OR "ST MI"))
Cochrane Library	Dual anti-platelet therapy AND Acute coronary syndrome AND platelet function test*

**Embase (Ovid)**	((dual anti-platelet therapy or dual antiplatelet) and Platelet function testing and acute coronary syndrome).mp. [mp=title, abstract, heading word, drug trade name, original title, device manufacturer, drug manufacturer, device trade name, keyword, floating subheading word, candidate term word]
**Google Scholar**	"Acute Coronary Syndrome" AND "Platelet Function Testing" OR "Platelet Function Tests" AND "Anti-platelet therapy" OR "antiplatelet therapy" OR "dual anti-platelet therapy" AND "P2Y12 antagonists" AND "Major adverse cardiac events" AND "Bleeding events"

Supplementary Table 2. Characteristics of included studies.

Study		Samardzic et al. 2014	Wang et al. 2015 (TRANSLATE-POPS)	Cayla et al. 2016 (ANTARCTIC)	Sibbing et al. 2017 (TROPICAL-ACS)	You et al. 2020 (PATROL)
Total no. of patients in each group	Guided Therapy	43	1,987	435	1,304	105
	Standard Therapy	44	1,830	442	1,306	195
Clinical Presentation n (%)	STEMI	Standard arm- 28 (63.6%)<br><br>Guided arm- 25 (58.1%)	Standard arm-52.7%<br><br>Guided arm- 49.8%	Standard arm- 150 (34%)<br><br>Guided arm - 152 (35%)	Standard arm- 722 (55%)<br><br>Guided arm- 731 (56%)	100%
	NSTEMI	Standard arm- 10 (22.7%)<br><br>Guided arm- 10 (23.3%)	-	Standard arm- 220 (50%)<br><br>Guided arm - 198 (46%)	Standard arm- 584 (45%)<br><br>Guided arm - 573 (44%)	-

UA	Standard arm- 6 (13.7%)<br><br>Guided arm- 8 (18.6%)	-	Standard arm- 72 (16%)<br><br>Guided arm- 85 (20%)	-	-
ACS	100%	100%	100%	100%	100%
PCI	YES	YES	YES	YES	YES
Assays and cutoff values	Multiplate analyser assay; HPR >46 U	VerifyNow P2Y12 assay; PRU ≥235	VerifyNow P2Y12 assay; HPR ≥208 PRU & LPR ≤85 PRU	Multiplate analyser assay; HPR ≥ 46 Units	Platelet VASP assay; HPR ≥ 50%
Randomisation & PFT Timepoints	Randomisation – After PCI<br><br>PFT – 12 to 24 hours after PCI	Randomisation – after PCI<br><br>PFT – Before discharge at least 12	Randomisation – Before allocation to study arms after PCI<br><br>PFT – Before treatment adjustment,	Randomisation – Before hospital discharge<br><br>PFT – 14 days post-discharge treatment	Randomisation – after PCI insertion<br><br>PFT – 72 hours after PCI

			hours post-PCI	14 days after treatment adjustment & at day 28		
P2Y12 antagonist + add-on drugs	Guided Therapy Group	Up to 2× clopidogrel 600mg LD + (75-300mg) MD	75mg clopidogrel 10mg prasugrel ticagrelor MD	INITIALLY<br><br>PR within range – continued prasugrel 5mg<br><br>HPR – prasugrel 10mg<br><br>LPR – prasugrel 5mg to clopidogrel 75mg<br><br>AFTER 14 DAYS	One week: 5mg/10mg prasugrel<br>&<br>One week: 75mg clopidogrel<br><br>THEN<br><br>5mg/10mg prasugrel MD (HPR)<br><br>75mg clopidogrel MD (non-LPR)	90mg ticagrelor

			LPR (10mg prasugrel) – 5mg prasugrel<br><br>LPR (75mg clopidogrel) – no further change<br><br>HPR (75mg clopidogrel) –5mg prasugrel<br><br>HPR (10mg prasugrel) – no further modification		
Standard Therapy Group	75mg clopidogrel MD	75mg clopidogrel 10mg prasugrel ticagrelor MD	5mg prasugrel MD	5mg/10mg prasugrel MD	75mg clopidogrel MD

Guided Strategy	Escalation	De-escalation & Escalation	De-escalation & Escalation	De-escalation	Escalation
Follow-up duration	12 months; 24 months	30 days; 12 months	12 months	12 months	12 months

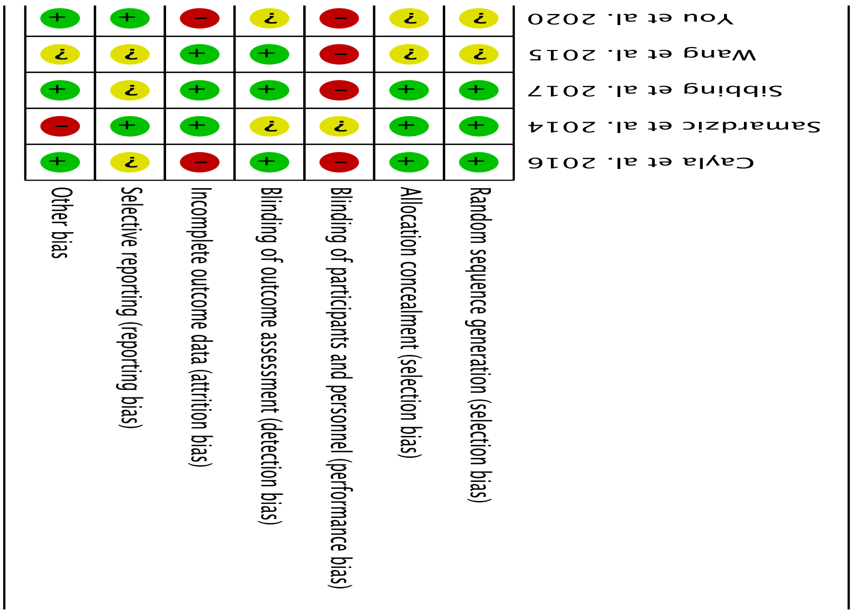

Supplementary Figure 1. Summary table of review authors' judgements for each risk of bias item. The five studies included in the meta-analysis were assessed for risk of bias against six definite bias domains. The "other bias" domain captured differences in baseline characteristics between the treatment arms. Green and "+" represents no bias, yellow and "?"unclear whether bias present, and red and "-" bias present.

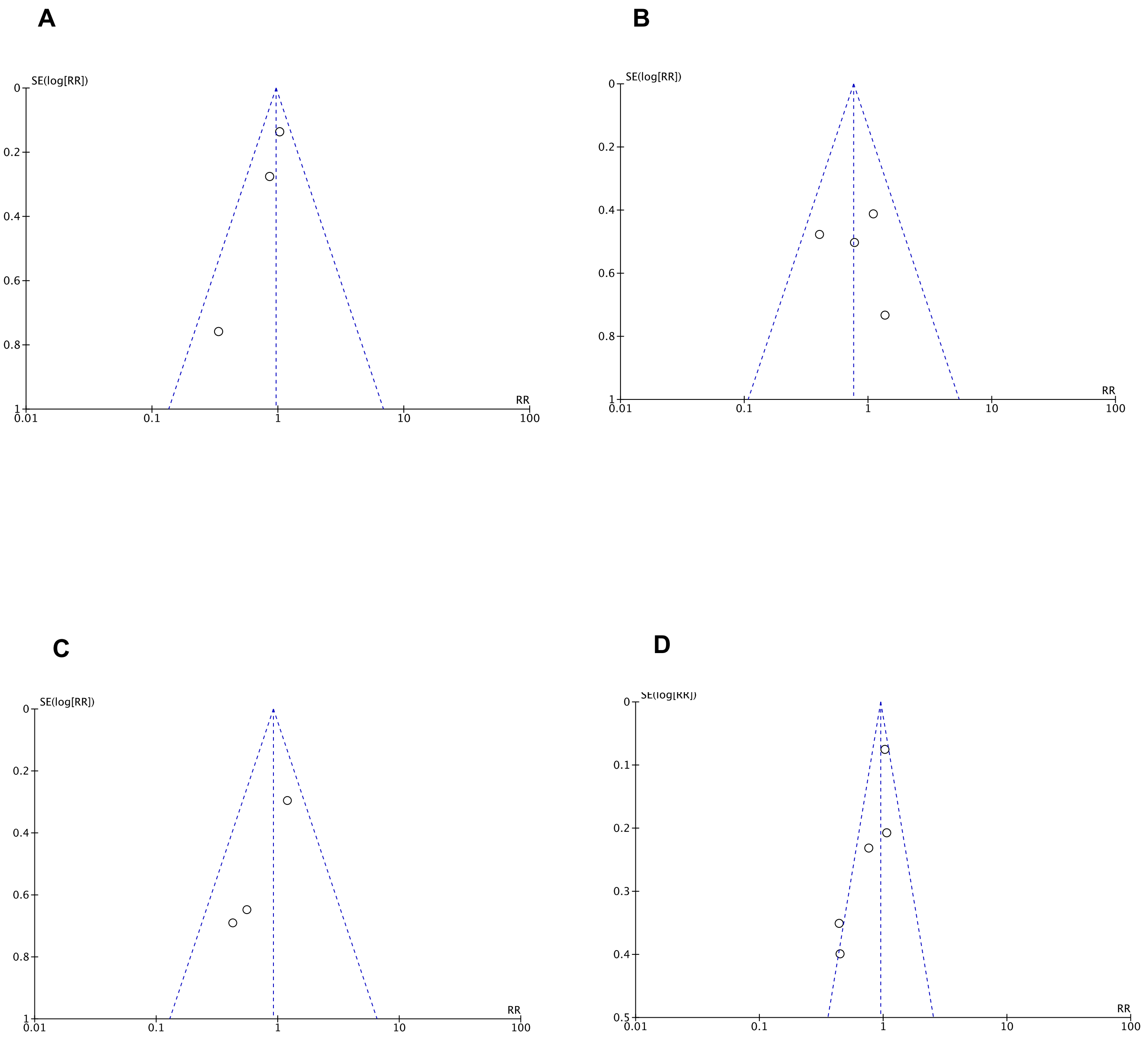

Supplementary Figure 2. Funnel plots for MI (**A**), cardiovascular death (**B**), stroke (**C**) and MACE (**D**).

Study or Subgroup	PFT Guided Therapy Events	Total	Standard Therapy Events	Total	Weight	Risk Ratio M-H, Fixed, 95% CI
Cayla et al. 2016	43	435	41	442	10.8%	1.07 [0.71, 1.60]
Samardzic et al. 2014	7	43	16	44	4.2%	0.45 [0.20, 0.98]
Wang et al. 2015	317	1987	283	1830	78.0%	1.03 [0.89, 1.20]
You et al. 2020	9	105	38	195	7.0%	0.44 [0.22, 0.87]
**Total (95% CI)**		**2570**		**2511**	**100.0%**	**0.97 [0.85, 1.11]**
Total events	376		378			

Heterogeneity: $Chi^2 = 9.73$, df = 3 (P = 0.02); $I^2 = 69\%$
Test for overall effect: Z = 0.46 (P = 0.64)

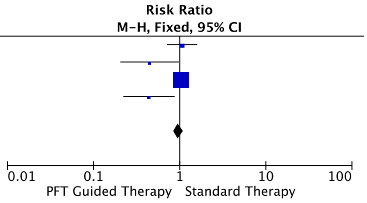

Supplementary Figure 3. Forest plot for MACE, having excluded the TRANSLATE-POPS study.
